# Sex difference in the interrelationship between TNF-α and oxidative stress status in first-episode drug-naïve schizophrenia

**DOI:** 10.1186/s12974-021-02261-5

**Published:** 2021-09-15

**Authors:** Minghuan Zhu, Zhenjing Liu, Yanhong Guo, Mst. Sadia Sultana, Kang Wu, Xiaoe Lang, Qinyu Lv, Xiao Huang, Zhenghui Yi, Zezhi Li

**Affiliations:** 1 Shanghai Mental Health Center, Shanghai Jiao Tong University School of Medicine, 600 South Wan Ping Road, Shanghai, 200030 China; 2 Clinical Research Center for Mental Disorders, Shanghai Pudong New Area Mental Health Center, School of Medicine, Tongji University, Shanghai, China; 3 Qingdao Mental Health Center, Qingdao University, Qingdao, China; 4 Department of Public Health and Informatics, Jahangirnagar University, Savar, Dhaka, Bangladesh; 5 Department of Laboratory Medicine, Shanghai Changhai Hospital, Shanghai, China; 6 Department of Psychiatry, The First Clinical Medical College, Shanxi Medical University, Taiyuan, China; 7 Department of Psychological Medicine, Zhongshan Hospital, Fudan University, 180 Fenglin Road, Shanghai, 200032 China; 8 Department of Psychiatry, The Affiliated Brain Hospital of Guangzhou Medical University, 36 Mingxin Road, Guangzhou, 510370 China

**Keywords:** Sex difference, Schizophrenia, TNF-α, Oxidative stress, Interaction

## Abstract

**Background:**

Increasing evidence indicates that dysregulated TNF-α and oxidative stress (OxS) contribute to the pathophysiology of schizophrenia. Additionally, previous evidence has demonstrated sex differences in many aspects of schizophrenia including clinical characteristics, cytokines, and OxS markers. However, to the best of our knowledge, there is no study investigating sex differences in the association between TNF-α, the OxS system, and their interaction with clinical symptoms in schizophrenia patients, especially in first-episode drug-naïve (FEDN) patients.

**Methods:**

A total of 119 FEDN schizophrenia patients and 135 healthy controls were recruited for this study. Serum TNF-α, superoxide dismutase (SOD), glutathione peroxidase (GSH-Px), catalase (CAT), and malondialdehyde (MDA) were measured. The Positive and Negative Syndrome Scale (PANSS) was applied to evaluate psychotic symptoms. Two-way ANOVA, partial correlation analysis, and multivariate regression analysis were performed.

**Results:**

A sex difference in MDA levels was demonstrated only in healthy controls (*F* = 7.06, *p*_Bonferroni_ = 0.045) and not seen in patients. Furthermore, only male patients had higher MDA levels than male controls (*F* = 8.19, *p*_Bonferroni_ = 0.03). Additionally, sex differences were observed in the association of TNF-α and MDA levels with psychotic symptoms (all *p*_Bonferroni_ < 0.05). The interaction of TNF-α and MDA was only associated with general psychopathology symptom in male patients (*B* = − 0.07, *p* = 0.02).

**Conclusion:**

Our results demonstrate the sex difference in the relationship between TNF-α, MDA, and their interaction with psychopathological symptoms of patients with schizophrenia.

## Introduction

Schizophrenia is a chronic and severe mental disorder characterized by psychopathological symptoms. The exact mechanisms of schizophrenia are still unclear [1]. Growing evidence suggests that the etiology of schizophrenia may be associated with dysregulated inflammatory pathways and oxidative stress (OxS) [2–4].

The activation of the inflammatory system as seen in cytokine activity may be closely related to susceptibility to schizophrenia [5–7]. TNF-α is one of the most important pro-inflammatory cytokines and contributes heavily to the pathophysiological process of schizophrenia [8, 9]. The abnormal expression of TNF-α pathway in schizophrenia patients has been well documented in the existing literature [10, 11]. OxS also plays an important role in the pathogenesis of schizophrenia [12]. Cadet and Lohr firstly suggested the role of oxidative mechanisms in schizophrenia in the 1980s [13] and noted the role of oxidative stress in brain dopamine (DA) systems, which are involved in the pathogenesis of schizophrenia [14]. Then, amounting studies have subsequently documented increased OxS and oxidative injury as well as an impaired antioxidant defense system in patients with schizophrenia [15, 16], such as superoxide dismutase (SOD), glutathione peroxidase (GSH-Px), catalase (CAT), and malondialdehyde (MDA). The dopamine system has been one of the most enduring and central hypotheses of schizophrenia. In neurons, DA can be auto-oxidized and causes the production of ROS including DA-quinones and superoxide [17, 18], which was supposed to be involved in the pathophysiology of schizophrenia [19]. On the other hand, previous evidence has suggested that spontaneous abnormal involuntary movements, a part of the symptoms of schizophrenia, might be associated with the pathophysiology of the disease itself without exposure to antipsychotics [20, 21]. The course and the progression of schizophrenia may share the same process with the development of abnormal movements [22].

In addition, it is worthy of note that the OxS system plays a central role through its interaction with the inflammatory system [23]. Reciprocal interactions between OxS and inflammatory systems have been established in previous studies [24, 25]. Previous research has revealed that the mechanism of certain brain developmental disorders caused by the activation of maternal immune systems may be closely related to OxS [26]. The activation of immune cells can secrete OxS mediators, while OxS mediators can also activate and enhance various inflammatory molecules and the immune responses [27]. Additionally, a corresponding relationship between the intensity of immune response and the level of OxS in schizophrenia has been demonstrated in a previous study [28]. Therefore, the relationship between cytokines and OxS mediators must be taken into account. A recent meta-analysis showed that patients with first-episode psychosis (FEP) had lower total antioxidant status, but higher IL6 and TNF-α compared to controls [29]. Correspondingly, our previous studies have also found that TNF-α, the OxS system, and their interaction were involved in the pathophysiology of schizophrenia [30].

Another critical concern is that there are sex differences in many aspects of schizophrenia including incidence rate, onset age, symptoms severity, cognitive function, response to antipsychotics, comorbidities, and outcomes [31–36], which may be partly related to psychosocial factors and sex hormones [37]. Furthermore, there are sex differences in levels of cytokines and OxS markers in schizophrenia patients [38–40]. For example, Lee et al. demonstrated sex differences in cytokine biomarkers of schizophrenia patients [38], including TNF-α [39]. In addition, previous evidence has shown that female may be more protected against oxidative stress [40], and some preclinical studies have also observed sex differences in oxidative stress markers, including glutathione (GSH), nitrite level, and lipid peroxidation in the hippocampus or striatum in models of schizophrenia [41, 42]. However, other studies found no sex differences in a set of oxidative stress biomarkers, including antioxidant enzymes (GPX and SOD), and MDA levels in either chronic patients [43, 44] or first-episode schizophrenia patients when utilizing a small sample size [45, 46]. These inconsistent results might be attributable to different disease stages or antipsychotics exposure. The sex difference in TNF-α and the OxS system has not yet been adequately explored. In particular, we have determined to the best of our knowledge that there is no current study examining sex differences in the association between TNF-α, the OxS system, and their interrelationship with clinical symptoms in patients with schizophrenia. Thus, this study was undertaken to fill this important knowledge gap.

In this study, first-episode drug-naïve (FEDN) schizophrenia patients were recruited to investigate (1) sex differences in cytokine TNF-α and OxS parameters of FEDN schizophrenia patients and (2) sex differences in the association of TNF-α, the OxS system, and their interaction with clinical symptoms.

## Participants and methods

### Participants

The protocol for this study was reviewed and approved by Shanghai Mental Health Center and the First Hospital of Shanxi Medical University. Informed consent was obtained from all participants prior to participation in this study. Inclusion and exclusion criteria were detailed in our previous study [30]. Briefly, inclusion criteria included (i) being Han Chinese; (ii) aged from 18 to 45 years old; (iii) meeting diagnostic criteria for schizophrenia according to the Diagnostic and Statistical Manual of Mental Disorders, Fourth Edition (DSM-IV); (iv) being a first-episode patient without prior exposure to drugs; and (v) having a duration of illness less than 2 years. Exclusion criteria included (i) individuals with any other major Axis I disorder and (ii) pregnant women.

Healthy controls were recruited based on having no major Axis I disorder diagnosis and no family history of mental disorders. Moreover, participants who had organic brain diseases, ongoing infections, autoimmune disorders, other severe physical diseases, or who received any immunosuppressive treatments were excluded from this study. A total of 119 FEDN patients with schizophrenia and 135 healthy controls were recruited. The demographic data were detailed in our previous study [30]. There were no significant differences in age, sex, education, body mass index (BMI), or smoking behavior between patients and healthy controls [30].

### Clinical interview and assessments

The Structured Clinical Interview for DSM-IV Axis I Disorders-Patient Edition (SCID-I/*P*) was applied by two psychiatrists to screen the participants. Demographic and clinical data were collected by a self-designed questionnaire. The Positive and Negative Syndrome Scale (PANSS) was applied to evaluate psychotic symptoms. Inter-rater concordance of assessments was over 0.8.

### Peripheral blood sampling and serum biochemical assays

After fasting overnight for at least 12 h, peripheral venous blood samples of 5 ml volume were collected between 07:00 and 09:00 am. Serum was isolated and was stored at − 80 °C until the assays were performed. The levels of TNF-α, SOD, GSH-Px, CAT, and MDA were measured through Enzyme-linked immunosorbent assays (ELISAs) (R&D Systems, USA). The researchers conducted this experiment according to the manufacturer’s protocol and they were blind to clinical data of samples. All samples were run in duplicate. Random samples were measured to verify the reproducibility of the assay. The intra-assay and inter-assay coefficients of variation were 6.8–7.6% and 6.2–7.4%, respectively.

### Statistical analysis

The Kolmogorov-Smirnov test was applied to detect the distribution normality of variables. As serum TNF-α levels distribute non-normally, this measure was transformed to a natural logarithm. Either a Fisher’s exact test or chi-squared test was conducted for nominal variables. Analysis of variance (ANOVA) was conducted for continuous variables.

To investigate sex differences in TNF-α, SOD, GSH-Px, CAT, and MDA levels, two-way ANOVA (diagnosis × sex) was applied, with each index as a dependent variable, setting diagnosis, and sex as fixed factors and adjusting for confounding variables. The main effects of diagnosis, sex, and diagnosis × sex interaction were calculated in each model. Then, an analysis of covariance (ANCOVA) was applied to examine individual univariate effects.

To examine the association between each serum parameter with clinical psychotic symptoms in male and female patients, a partial correlation analysis was performed, controlling for age, BMI, smoking, education, and onset age. Furthermore, to investigate the association of the interaction between TNF-α and OxS parameters with clinical psychotic symptoms in male and female patients, multivariate regression analysis was performed. In this multivariate regression analysis, each PANSS total or subscale score was set as a dependent variable, each interaction (TNF-α × SOD, TNF-α × GSH-Px, TNF-α × CAT, and TNF-α × MDA) as an independent variable, and age, BMI, smoking, education, and onset age were adjusted as covariates. Multiple comparisons were corrected by Bonferroni corrections. Data were analyzed using SPSS version 23.0. The α level of significance was set to *p* < 0.05 (two-tailed).

## Results

### Sex difference in demography and clinical characteristics of patients

As shown in Table 1, there were significant sex and diagnosis × sex effects on education (both *p* < 0.001). An ANOVA demonstrated that male and female patients had lower education than male and female controls, respectively (*F* = 33.74, *p* < 0.001; *F* = 10.28, *p* = 0.002). Furthermore, male controls had higher education than female controls (*F* = 106.98, *p* < 0.001). Education was adjusted for in the following analysis. There was a significant diagnosis × sex effect on BMI (*F* = 7.44, *p* = 0.007). An ANOVA showed that male patients had a lower average BMI than female patients (*F* = 7.22, *p* = 0.008). BMI was adjusted for in the following analysis.

**Table 1:** Sex difference in demographic information between healthy controls and schizophrenia patients

	Controls		Patients		Diagnosis, *F* (*p*)	Sex, *F* (*p*)	Diagnosis × sex, *F* (*p*)
	Male (*n* = 80)	Female (*n* = 55)	Male (*n* = 76)	Female (*n* = 43)			
Age (years)	28.65 ± 7.63	29.69 ± 7.87	29.68 ± 6.96	28.83 ± 7.67	0.009 (0.93)	0.02 (0.92)	0.95 (0.33)
Education (years)^a,b,c^	13.92 ± 2.07	9.69 ± 2.65	11.55 ± 2.90	11.86 ± 3.98	0.07 (0.80)	28.37 (< 0.001)	37.99 (< 0.001)
BMI^d^	23.57 ± 3.62	22.72 ± 3.24	22.21 ± 2.92	23.71 ± 3.61	0.19(0.67)	0.56 (0.45)	7.44 (0.007)
Smoking^e^	8 (10.0%)	1 (1.8%)	11 (14.5%)	1 (2.3%)	-	-	-

2 × 2 ANCOVA was applied to compare sex difference in each variable

^a^Significant differences between male patients and male controls

^b^Significant differences between female patients and female controls

^c^Significant differences between male and female controls

^d^Significant differences between male and female patients

^e^Fisher’s exact test

As shown in Table 2, female patients had an earlier average onset age than male patients (*F* = 7.22, *p* = 0.01). Onset age was controlled in the following analysis. There were no significant differences in total score and subscale scores of PANSS between male and female patients (all *p* > 0.05).

**Table 2:** Clinical characteristics of male and female patients with schizophrenia

Variable	Male (*n* = 76)	Female (*n* = 43)	*F*	*p*
Onset age (years)	22.11 ± 7.69	19.47 ± 9.92	6.96	0.01
PANSS score				
Positive symptoms	19.15 ± 8.77	18.95 ± 8.43	0.93	0.34
Negative symptoms	26.24 ± 7.29	28.04 ± 7.36	1.35	0.25
General psychopathology	42.36 ± 8.88	40.47 ± 7.92	0.95	0.33
Total score	87.74 ± 15.38	87.47 ± 11.83	0.32	0.57

### Sex difference in levels of TNF-α and OxS parameters between patients and healthy controls

**Fig. 1:**
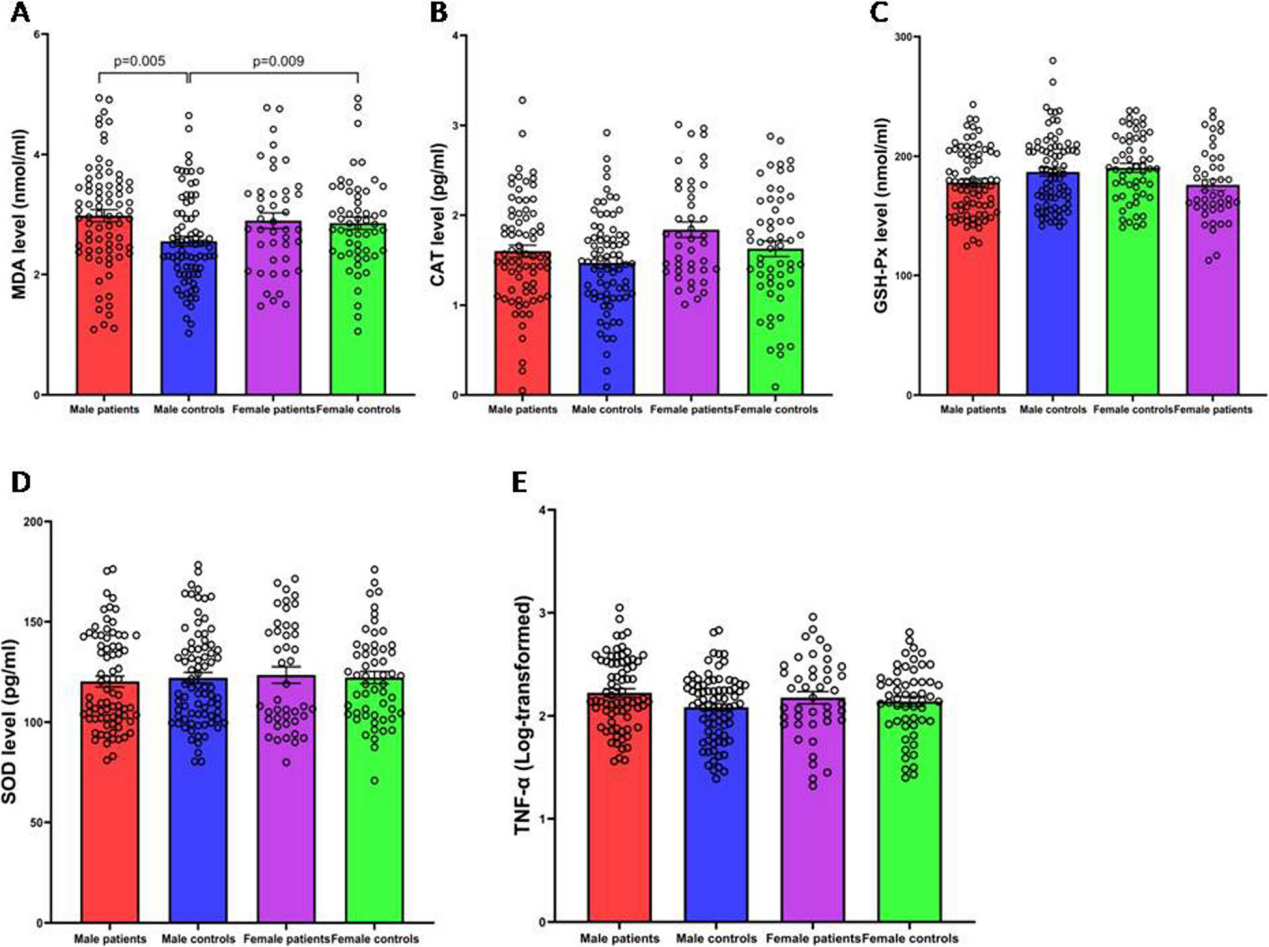
Sex difference in levels of SOD, GSH-Px, CAT, MDA, and TNF-α between patients and healthy controls (mean ± SEM). Male patients (*N* = 76), male controls (*N* = 80), female patients (*N* = 43), and female controls (*N* = 55). The two-way ANOVA adjusted for education and BMI showed significant main effects of diagnosis on GSH-Px (*F* = 9.09, *p* = 0.003), CAT (*F* = 4. 96, *p* = 0.03), MDA (*F* = 4.99, *p* = 0.03), and TNF-α (*F* = 4.15, *p* = 0.04), except SOD (*F* = 0.01, *p* = 0.92) (**A**–**E**). There was a significant diagnosis × sex effect on MDA levels (*F* = 3.78, *p* = 0.05). Then, ANCOVA showed that MDA levels were higher in female healthy controls than in male healthy controls (*F* = 7.06, *p* = 0.009, *p*_Bonferroni_ = 0.045), and that MDA levels were higher in male patients than in male healthy controls (*F* = 8.19, *p* = 0.005, *p*_Bonferroni_ = 0.03) (**A**). There was a main effect of sex on CAT levels indicating sex difference in CAT levels (*F* = 5.53, *p* = 0.02) (**B**). SOD, superoxide dismutase; GSH-Px, glutathione peroxidase; CAT, catalase; MDA, malondialdehyde

As shown in Fig. 1, a two-way ANOVA that was adjusted for education and BMI demonstrated a main effect of diagnosis on TNF-α, GSH-Px, CAT, and MDA (all *p* < 0.05), indicating differences in the levels of TNF-α, GSH-Px, CAT, and MDA between patients and healthy controls. There was a main effect of sex on CAT levels indicating sex difference in CAT levels (*F* = 5.53, *p* = 0.02). Also, there was a significant diagnosis × sex effect on MDA levels (*F* = 3.78, *p* = 0.05), indicating that sex differences in the levels of MDA observed were different between patients and controls. Further, ANCOVA showed that MDA levels were higher in female healthy controls than in male healthy controls (*F* = 7.06, *p* = 0.009, *p*_Bonferroni_ = 0.045) and that MDA levels were higher in male patients than in male healthy controls (*F* = 8.19, *p* = 0.005, *p*_Bonferroni_ = 0.03). There was no difference in levels of MDA between female patients and female healthy controls (*F* = 0.01, *p* = 0.92, *p*_Bonferroni_> 0.05).

### Differences in the relationship between TNF-α and OxS parameters and psychotic symptoms as categorized by sex

**Fig. 2:**
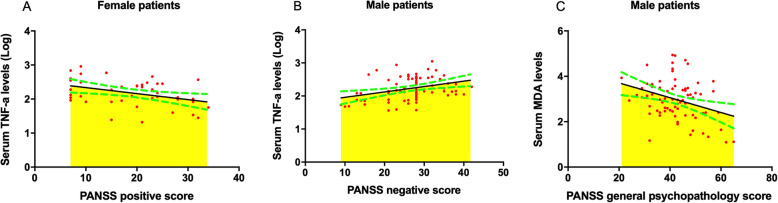
The association of TNF-α and MDA levels with psychotic symptoms categorized by sex. A partial correlation analysis was applied. The green dotted curve presents 95% confidence interval. **A** The association between TNF-α levels and positive symptoms in female patients with schizophrenia (*N* = 43). TNF-α levels were associated with PANSS positive score in female patients (*r* = − 0.49, *p* = 0.002, *p*_Bonferroni_= 0.008). **B** The association between TNF-α levels and positive symptoms in male patients with schizophrenia (*N* = 76). TNF-α levels were associated with PANSS negative score in male patients (*r* = 0.31, *p* = 0.01, *p*_Bonferroni_= 0.04). **C** The association between MDA levels and general psychopathology symptom in male patients with schizophrenia (*N* = 76). MDA levels were associated with PANSS general psychopathology scores in male patients (*r* = − 0.32, *p* = 0.007, *p*_Bonferroni_= 0.03)

As shown in Fig. 2A, controlling for the covariates age, BMI, education, smoking, and onset age, partial correlation showed that TNF-α levels were associated with PANSS positive score in female patients (*r* = − 0.49, *p* = 0.002, *p*_Bonferroni_= 0.008). However, there was no association of TNF-α levels with PANSS positive score in male patients (*r* = − 0.11, *p* = 0.36, *p*_Bonferroni_ > 0.05). As shown in Fig. 2B, partial correlation showed that TNF-α levels were associated with PANSS negative score in female patients (*r* = 0.37, *p* = 0.02) and in male patients (*r* = 0.31, *p* = 0.01). However, after Bonferroni correction, significance remained only for male patients (*p*_Bonferroni_= 0.04). As shown in Fig. 2C, MDA levels were associated with PANSS general psychopathology scores in male patients (*r* = − 0.32, *p* = 0.007, *p*_Bonferroni_= 0.03), but no association in female patients (*r* = 0.02, *p* = 0.92, *p*_Bonferroni_> 0.05) was found.

### Differences in the relationships of TNF-α and OxS interactions with psychotic symptoms as categorized by sex

To examine sex differences in the association of interaction between TNF-α × SOD, TNF-α × GSH-Px, TNF-α × CAT, or TNF-α × MDA with psychotic symptoms, multivariate regression analysis was applied in male and female patient populations, separately. After controlling for the covariates age, BMI, education, smoking, and onset age, multivariate regression analysis showed that TNF-α × MDA was associated with PANSS general psychopathology scores in male patients (*B* = − 0.07, *t* = 2.46, *p* = 0.02) but not in female patients (*B* = 0.03, *t* = 0.81, *p* = 0.43). Moreover, there was no association of interaction between TNF-α × SOD, TNF-α × GSH-Px, or TNF-α × CAT with any PANSS subscale or total score (all *p* > 0.05).

## Discussion

To the best of our knowledge, this study was the first to examine sex differences in TNF-α, OxS, and their interactions in FEDN schizophrenia patients. The main findings of this study were as follows: (i) There was no sex difference in psychopathology symptoms of the patients. (ii) There were sex differences in MDA levels of healthy controls, but not of schizophrenia patients. MDA levels of female controls were higher than those of male controls, but MDA levels of female patients were similar to those of male patients. (iii) There were sex differences in the association between TNF-α and MDA levels and psychotic symptoms. The interaction between TNF-α and MDA correlated with general psychopathology symptom in male patients only.

Our previous study has shown that FEDN patients had higher TNF-α and MDA levels than in healthy controls. Amounting evidence has revealed the activated TNF pathway in schizophrenia [11]. The immune-neurotoxicity of peripheral TNF-α is associated with psychotic symptoms and cognitive deficits of schizophrenia patients [47]. There are several paths for exchanging cytokines between the periphery and CNS, such as through leakage of the blood-brain barrier (BBB), vagus afferents, and cross-talk between peripheral circulation and central nervous system [48–51]. Furthermore, previous clinical and preclinical studies have shown that prenatal exposure to infection increased the risk of schizophrenia, and TNF-α decreased the nodes, total dendritic length and inhibit cortical neuron dendrite development, which suggested that brain TNF-α could impair neuronal survival and development [52, 53]. However, the increased TNF-α in the periphery and the brain which acts through indirect and direct pathways may be complex. Klaus et al. reported that both peripheral and brain region-specific increases in TNF could cause abnormal behaviors through different pathways [54]. In addition, dopamine metabolism, involved in the pathophysiology of schizophrenia, is strongly associated with oxidative stress due to its degradation and autooxidation [55]. Besides oxidative stress, DAergic neurons can also release chemoattractants, both of which can lead to microglia activation and inflammatory responses [55].

In this study, we found no sex differences in psychopathological symptoms of FEDN patients, which was consistent with previous studies [56, 57]. However, our results regarding sex differences in symptoms of schizophrenia were not consistent with the current literature. Several studies have shown that males have more negative symptoms than females [58]. González-Rodríguez et al. [59] pointed out that differences in methodology, sample size, and a lack of a systematic and homogenous assessment of psychopathological symptoms may have contributed to the observed discrepancies.

We found that MDA levels of female controls were higher than those of male controls, but those female patients had the same level of MDA as male patients. At present, the results of studies on the differences in MDA levels between men and women are not yet in agreement. However, Kharb et al. [60] found that female healthy controls had higher serum MDA levels than male healthy controls, which corresponds to our findings. Consistent to our findings, several studies have demonstrated no sex difference in MDA levels of first-episode schizophrenia [61] or chronic schizophrenia patients who received stable antipsychotic drugs [62]. The possible reason may be attributable to the following reasons. First, testosterone is an important male sex hormone secreted mainly by male testes. Previous studies have found that testosterone has the effect of antioxidative stress [63, 64]. Wang et al. [65] reported that testosterone supplementation significantly decreased the concentration of MDA in the hippocampus, which explains the higher MDA levels observed in female controls when compared to male healthy controls. Second, in regards to the patients with schizophrenia, increased dopamine in the nigra-striatal pathway is considered to be a driving force of psychosis [66, 67], and the effectiveness of antipsychotics that block the dopamine D2 receptor in relieving hallucinations and delusions is well established [68]. One of the direct dopamine agonists, amphetamine stimulates the release of dopamine [69, 70] and has been reported to inhibit testosterone release in male rats [71, 72]. This suggests that hyperfunction of the dopamine system in schizophrenia patients may inhibit the release of testosterone, which may explain the reasons for having no sex difference in the level of MDA in schizophrenia patients in comparison to controls. Interestingly, Qu et al. [48] recently found that in healthy controls, women had lower MDA levels than men. This contrary result regarding MDA levels in male and female healthy controls might be explained by the characteristics of the samples used. For example, male and female healthy controls had significant differences in age and BMI in that study, which may have influenced the results. In agreement to this, previous reports have shown that oxidative stress is associated with aging and BMI [73]. The level of reactive oxygen species (ROS) increases with the advancement of age [74] and was associated with BMI [75]. In addition, unlike the result of MDA, there was a main effect of sex on CAT levels, but no diagnosis × sex effect indicating sex difference in CAT levels in both patients and controls.

Previous evidence has shown a strong interaction between OxS and the inflammatory system. Astrocytes and microglia can be activated by OxS, causing inflammatory response dysfunction, while the OxS system in nonphagocytic cells can be activated by cytokines including TNF-α [76, 77]. Buelna-Chonta et al. pointed out that the complicated interaction between inflammation and OxS is partly determined by the interaction between the transcription factor Nf-kappaB with Nrf2 [78]. Moreover, previous evidence suggested that neuroinflammation and persistent OxS are critical aspects in the pathophysiology of neurodegenerative diseases [77]. Because of the close relationship between these two systems, Steullet et al. [23] believed that the neuroimmune system, OxS, and glutamatergic system constitute a “central hub,” and that the disturbance of these “hub” systems may lead to the abnormality of parvalbumin interneurons and white matter in patients with schizophrenia through the dysfunction of macro-circuits and micro-circuits. This dysfunction, in turn, affects the symptoms of patients. However, the interaction of TNF-α and OxS on the susceptibility and clinical characteristics of schizophrenia has not been investigated well. We previously reported that the interaction between TNF-α and MDA increased the risk for the occurrence of schizophrenia by 1.61 times, but no significant interactive effects were found on any domain of the PANSS [30]. A recent study also showed that TNF-α was associated with lowered IgM/MDA [47]. In this study, after patients were stratified by sex, we found that the interaction between TNF-α and MDA activities was associated with the severity of general psychopathology in male schizophrenia patients, suggesting that TNF-α and MDA have an interactive effect on the psychopathological symptoms only in male patients. The possible mechanisms may also be associated with testosterone, which can affect the MDA expression. Additionally, testosterone can also affect the expression of inflammatory markers, including TNF-α. Preclinical studies have shown that high testosterone levels during embryonic development have adverse effects on immune function [79]. Furthermore, the use of testosterone significantly reduced the level of inflammatory markers in men [80]. It has been found that the level of TNF-α is higher in adult men with lower testosterone levels [81], while the expression of TNF-α is inhibited by testosterone in men with hypogonadism [82]. A study conducted by Delfino et al. pointed out that TNF-α and NF-kappaB, which may be involved in the interaction between oxidative stress and inflammation [78], could stimulate the expression of androgen receptors in Sertoli cells, and this may be an important mechanism for increasing the response of Sertoli cells to testosterone [83]. This suggests that androgen may have complex interactions with the immune system and OxS, which may explain the reasons for the interaction existence between TNF-α and MDA only in male FEDN schizophrenia patients. Moreover, there were sex differences in the association between TNF-α and MDA levels and psychotic symptoms. The underlying mechanisms should be further investigated in future studies.

Interestingly, there are sex differences in the prevalence of Parkinson’s disease, a disorder that also involves dopaminergic neuron, which is also mainly due to sex hormones [84, 85]. Most importantly, it is well established that methamphetamine exposure can lead to psychotic syndrome similar to schizophrenia mainly through dopaminergic neurotransmission, and repeated methamphetamine administration was used to build an animal model of schizophrenia [86, 87]. Furthermore, oxidative stress and inflammation may both play an important role in the pathophysiology of methamphetamine-associated psychosis [88]. On the other hand, there were also sex-dependent differences in methamphetamine exposure and toxicity [89, 90]. For example, Daiwile et al. demonstrated the sex difference in behavior and gene expression induced by methamphetamine exposure [91].

There were several limitations in this study. Firstly, it is not clear whether peripheral levels of TNF-α and OxS parameters are related to levels present in the brain. However, previous studies suggested that brain immune cells can monitor the peripheral innate immune response through a variety of parallel pathways, including afferent nerves, the humoral pathway, cytokine transporters at the blood-brain barrier, and IL-1 receptors on microvascular cells of the cerebral vein [92]. In addition, central neurons are highly sensitive to OxS exposure, and peripheral OxS can affect the activation of OxS response in brain neurons [93]. There are also extensive interactions between OxS and some other cytokines which should be investigated in future studies.

## Conclusion

Our results support the presence of sex differences in the association between TNF-α, MDA, and their interaction with psychopathological symptoms of patients with schizophrenia. Further study with a larger sample size should be conducted to validate our results.

## Data Availability

The data that support the findings of this study are available from the corresponding author Zezhi Li upon reasonable request.

## Abbreviations

ANOVA: Analysis of variance
ANCOVA: Analysis of covariance
BBB: Blood-brain barrier
BMI: Body mass index
CAT: Catalase
DA: Dopamine
DSM-IV: Diagnostic and Statistical Manual of Mental Disorders, Fourth Edition
ELISAs: Enzyme-linked immunosorbent assays
FEDN: First-episode drug-naïve
FEP: First-episode psychosis
GSH: Glutathione
GSH-Px: Glutathione peroxidase
MDA: Malondialdehyde
OxS: Oxidative stress
PANSS: Positive and Negative Syndrome Scale
ROS: Reactive oxygen species
SCID-I/*P*: Structured Clinical Interview for DSM-IV Axis I Disorders-Patient Edition
SOD: Superoxide dismutase
